# Comparative Efficacy of Platelet-Rich Plasma, Autologous Serum, and Artificial Tears in Dry Eye Disease: A Systematic Review and Meta-Analysis

**DOI:** 10.3390/biomedicines13092316

**Published:** 2025-09-22

**Authors:** Alexandra Laura Mederle, Diana Andrei, Laura Andreea Ghenciu, Emil Robert Stoicescu, Roxana Iacob, Ovidiu Alin Haţegan

**Affiliations:** 1 Department XIV, Discipline of Dermatology, “Victor Babeș” University of Medicine and Pharmacy, 300041 Timisoara, Romania; alexandra.mederle@umft.ro; 2 Department XVI, Discipline of Medical Rehabilitation, “Victor Babeș” University of Medicine and Pharmacy, 300041 Timisoara, Romania; 3 Department III, Discipline of Pathophysiology, “Victor Babeș” University of Medicine and Pharmacy, 300041 Timisoara, Romania; 4 Center for Translational Research and Systems Medicine, “Victor Babeș” University of Medicine and Pharmacy, 300041 Timisoara, Romania; 5 Radiology and Medical Imaging University Clinic, “Victor Babes” University of Medicine and Pharmacy Timisoara, Square Eftimie Murgu 2, 300041 Timisoara, Romania; stoicescu.emil@umft.ro; 6 Research Center for Pharmaco-Toxicological Evaluations, “Victor Babes” University of Medicine and Pharmacy Timisoara, Square Eftimie Murgu 2, 300041 Timisoara, Romania; 7 Field of Applied Engineering Sciences, Specialization Statistical Methods and Techniques in Health and Clinical Research, Faculty of Mechanics, “Politehnica” University Timisoara, Mihai Viteazul Boulevard No. 1, 300222 Timisoara, Romania; 8 Department of Anatomy and Embriology, “Victor Babes” University of Medicine and Pharmacy Timisoara, Square Eftimie Murgu 2, 300041 Timisoara, Romania; roxana.iacob@umft.ro; 9 Discipline of Anatomy and Embriology, Medicine Faculty, “Vasile Goldis” Western University of Arad, Revolution Boulevard 94, 310025 Arad, Romania; hategan.ovidiu@uvvg.ro

**Keywords:** dry eye disease, Sjogren’s syndrome, Schirmer test, OSDI, TBUT, artificial tears, autologous serum, PRP

## Abstract

**Background/Objectives**: Dry eye disease (DED) is a prevalent, complex disorder with a major impact on patients’ quality of life. While artificial tears (AT) are still the first-line treatment, their effectiveness is often limited in moderate-to-severe cases. Autologous serum (AS) and platelet-rich plasma (PRP) are now recognized as viable biologic treatments due to their regenerative and anti-inflammatory characteristics. This systematic review and meta-analysis sought to assess and compare the clinical efficacy of PRP, AS, and AT in the treatment of DED, with a focus on comparative studies. **Methods**: A comprehensive search of PubMed, Scopus, and Google Scholar was conducted until June 2025 for studies directly comparing PRP, AS, and AT. Eligible trials included patients with DED who reported results such as the Schirmer test, tear break-up time (TBUT), and Ocular Surface Disease Index (OSDI). The risk of bias was calculated using ROB 2 for randomized trials and ROBINS-I for non-randomized studies. Meta-analyses were carried out using standardized mean differences (SMDs) and 95% confidence intervals (CIs). **Results**: Seventeen studies were included in the systematic review. Both PRP and AS demonstrated greater improvements in OSDI, TBUT, and Schirmer test scores compared to AT. PRP showed a trend toward better outcomes than AS, especially in studies using injectable PRP. However, substantial heterogeneity and methodological variability were noted. **Conclusions**: Comparative research suggests that PRP and AS are more effective than AT in treating DED. Direct comparisons of PRP and AS yield varied results, with the route of delivery impacting outcomes. Given the heterogeneity of current protocols, further standardized, long-term trials are required to confirm the optimal delivery method and ensure safety.

## 1. Introduction

Dry eye disease (DED) is a multifactorial disorder of the ocular surface characterized by tear film instability and hyperosmolarity, inflammation, and damage to the ocular surface, often accompanied by visual disturbance and discomfort [1]. The prevalence of this disease varies widely throughout the world and is estimated to be between 5 and 50%, affecting quality of life, especially in the elderly [2]. Dry eye disease manifests through a wide range of symptoms, including dryness, itching, photophobia, foreign body sensation, burning, excessive tearing, and blurred vision, and in advanced cases, it can cause corneal ulcers or impaired visual acuity; persistent discomfort may also lead to increased anxiety, depression, and sleep disorders, profoundly affecting the patient’s psychological, social, and economic quality of life [3,4].

Standard treatment typically begins with artificial tears (AT), which aim to restore tear film volume and improve symptoms [5,6]. Nonetheless, these treatments frequently have temporary effects, making sustained control of chronic inflammatory responses on the ocular surface difficult. Furthermore, preservatives contained in many AT products could damage the ocular surface and potentially worsen symptoms, restricting their potential for prolonged use [7]. Preservatives such as benzalkonium chloride can exacerbate ocular surface inflammation and toxicity with frequent use [8]. Preservative-free artificial tears (PFATs) reduce this risk and are better tolerated, making them a safer long-term option for maintaining tear film stability and alleviating symptoms in sensitive or chronic cases [9,10].

In cases of moderate-to-severe DED, particularly those refractory to artificial tears, more advanced therapies are often necessary [11]. Among these, blood-derived eye drops are group of biologically based therapies prepared from the patient’s own blood and designed to mimic or replace the natural tear film. They have emerged as promising alternatives due to their biological similarity to natural tears and their content of growth factors and anti-inflammatory mediators [12]. Unlike AT, which mainly act as lubricants, blood-derived formulations provide bioactive components naturally present in healthy tears. These include growth factors such as epidermal growth factor, transforming growth factor-β, platelet-derived growth factor, and vascular endothelial growth factor, along with vitamins, fibronectin, albumin, and anti-oxidative enzymes, which together support epithelial proliferation, anti-inflammatory signaling, and ocular surface repair [13]. Furthermore, blood-derived therapies have been investigated to treat epithelial detachments, corneal ulcers, recurrent erosions, chemical corneal toxicity or limbal stem cell deficiency (LSCD) [14,15]. Blood-derived therapies include autologous serum and platelet preparations such as platelet-rich plasma (PRP), plasma rich in growth factors (PRGF), and platelet lysate, all of which include higher quantities of growth factors and cytokines, which lead to cellular repair and angiogenesis, aiding in wound repair and tissue regeneration [16]. The compositional differences between these blood-derived therapies underpin distinct biological effects and may influence therapeutic outcomes in DED [13]. While autologous serum has been demonstrated to be more efficient in treating severe dry eye illness than PFATs, the presence of circulating antibodies and pro-inflammatory cytokines may restrict its efficacy [17]. Despite their promise, blood-derived products are still expensive, problematic and varied, with few randomized clinical studies, variable diagnostic criteria, and no international standardization or regulatory consensus. Allogeneic blood products have recently emerged as a viable alternative that is unaffected by the patient’s health status, while their position in clinical practice is still evolving [18,19].

This systematic review and meta-analysis aimed to evaluate and compare the efficacy of PRP, AS, and AT in the treatment of DED. Given the growing interest in biologic therapies and the expanding use of PRP and AS as advanced alternatives to conventional lubricants, it is essential to assess their relative effectiveness. Comparative studies shed light on the therapeutic hierarchy of these treatments, directing medical professionals toward more evidence-based, customized DED care options. By focusing on head-to-head studies, this study addresses the existing gap in directly comparative evidence and helps clarify the role of biologics against that of conventional treatments in clinical practice.

## 2. Materials and Methods

### 2.1. Search Strategy and Study Selection

This review aimed to evaluate the efficacy of blood-derived eye drops, AS and PRP, in comparison to PFATs and conventional artificial tears for the treatment of DED. A comprehensive literature search was performed to identify studies that directly compared any two of the following treatments for DED: PRP, AS, and AT. The search covered a wide range of interventional studies, including randomized controlled trials and non-randomized comparative studies, without restrictions on publication date, setting, or language. The search strategy included keywords and combinations such as “dry eye,” “autologous serum,” “platelet-rich plasma,” “PRP,” “artificial tears,” “preservative-free artificial tears,” and “blood-derived eye drops.” In addition to database searching, the reference lists of relevant articles and previously published reviews were screened manually to identify any additional eligible studies. The search was performed independently by two authors.

Electronic databases searched included PubMed, Scopus, and Google Scholar, from inception to June 2025. The search combined keywords and medical subject headings (MeSH) related to “dry eye”, “platelet-rich plasma”, “autologous serum”, “artificial tears”, and “comparative studies”. Additional studies were identified by manually screening the reference lists of relevant articles and prior reviews.

**Figure 1:**
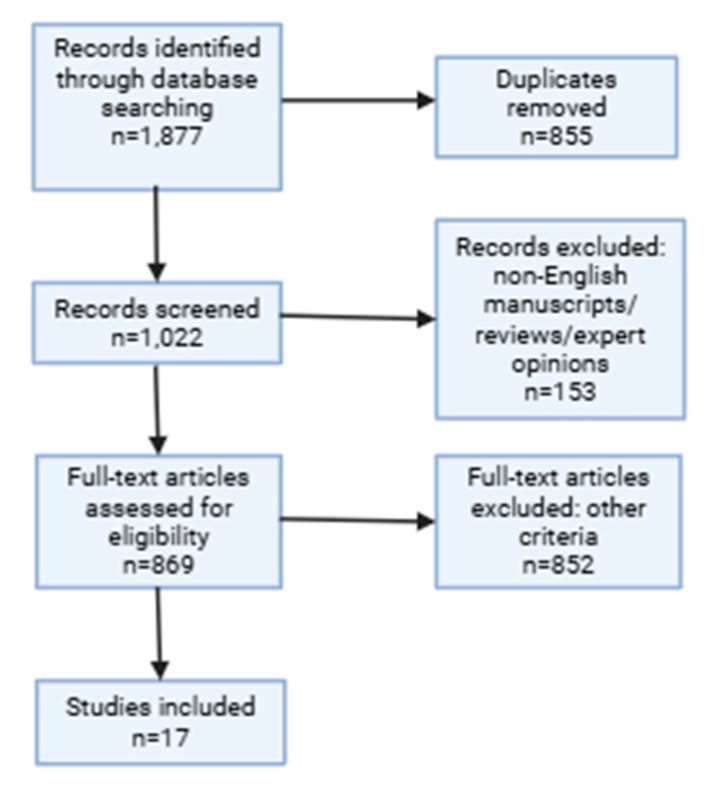
PRISMA 2020 flow diagram illustrating the study selection process. The diagram shows the number of records identified through database searching and other sources, the number of duplicates removed, the number of records screened, full-text articles assessed for eligibility, and the final number of studies included in the qualitative synthesis.

This systematic review was conducted in accordance with the Preferred Reporting Items for Systematic Reviews and Meta-Analyses (PRISMA) 2020 guidelines [20]. A PRISMA flow diagram was prepared to illustrate the study selection process, including identification, screening, eligibility, and inclusion of studies (Figure 1). The review protocol was registered with the International Prospective Register of Systematic Reviews (PROSPERO, registration number 1108353).

### 2.2. Eligibility Criteria

Studies were eligible for inclusion if they involved a direct comparison between any two of the following treatments: PRP, AS, or AT. Eligible studies enrolled patients with a clinical diagnosis of dry eye disease, regardless of underlying etiology. To qualify, studies had to report at least one of the following clinical outcomes relevant to dry eye disease: Ocular Surface Disease Index (OSDI), Schirmer test or tear break-up time (TBUT or NIBUT). Comparative interventional designs were accepted, including randomized controlled trials, prospective cohort studies, and retrospective comparative studies.

Studies were excluded if they did not include a comparative group (single-arm studies), evaluated combination therapies rather than head-to-head interventions, lacked extractable clinical outcome data, or were non-original research such as reviews.

### 2.3. Data Extraction and Outcomes

Data extraction was performed independently by two authors. Extracted information included study authorship, year and country of publication, study design, sample size, type of intervention, duration of follow-up and reported outcomes. The outcomes of interest included changes in OSDI scores, TBUT/NIBUT in seconds and Schirmer I test results in millimeters over five minutes. When standard deviations were not reported, they were estimated from available *p*-values or confidence intervals using assumptions appropriate for paired *t*-tests.

### 2.4. Risk of Bias Assessment

Risk of bias was evaluated using the Risk Of Bias In Non-randomized Studies-of Interventions (ROBINS-I) tool for non-randomized studies and the Cochrane RoB 2 tool for randomized controlled trials [21,22,23,24]. The ROBINS-I tool assesses seven domains of potential bias, including confounding, participant selection, classification of interventions, deviations from intended interventions, missing data, measurement of outcomes, and selection of reported results. Judgements were made for each domain, and an overall risk of bias was assigned as low, moderate, or high. The RoB 2 tool similarly addresses five domains tailored to randomized designs, focusing on randomization process, deviations from intended interventions, missing outcome data, measurement of the outcome, and selection of the reported results. For crossover trials, additional considerations were made for carryover effects and the adequacy of washout periods. Each study was categorized as having low risk, moderate risk, or high risk of bias based on these domains.

### 2.5. Statistical Analysis

For all comparative evaluations between AS, PRP, and AT, we extracted continuous outcome measures such as Schirmer test values, TBUT, and OSDI scores.

Effect sizes were calculated as standardized mean differences (SMDs) with 95% confidence intervals (CIs) for continuous outcomes, allowing for comparisons across studies using different measurement scales. To enhance comparability, we prioritized extracting and analyzing outcome data reported at similar follow-up durations across studies, when available. Heterogeneity was assessed using Cochran’s Q test and quantified with the I^2^ statistic, where values of 25%, 50%, and 75% corresponded to low, moderate, and high heterogeneity, respectively. A *p*-value < 0.10 for Q and an I^2^ > 50% were considered indicative of substantial heterogeneity.

Separate meta-analyses were conducted for each of the following pairwise comparisons: PRP vs. AS/PRP vs. AT. For each comparison, subgroup analyses were performed by outcome domain (Schirmer test, TBUT/NIBUT, OSDI score). If studies reported multiple follow-up points, we selected the longest available follow-up for inclusion in the meta-analysis. Studies that lacked sufficient data for pooling were included in the narrative synthesis only. All statistical tests were two-sided, and results were considered statistically significant at *p* < 0.05, except for tests of heterogeneity. Due to the limited number of studies per outcome (fewer than 10), formal statistical tests for publication bias, such as Egger’s test, were not performed, as they are underpowered in small samples.

For quantitative synthesis, forest plots were generated to visually display the effect sizes and 95% confidence intervals of individual studies and pooled estimates for each pairwise comparison (PRP vs. AS, PRP vs. AT, and AS vs. AT). Where applicable, outcomes were grouped and analyzed based on specific clinical parameters such as OSDI score, TBUT, and Schirmer test. Subgroup analyses were conducted to explore differences based on the route of administration and outcome domains.

## 3. Results

### 3.1. Overview of Studies

In total, 17 studies were included in the systematic review: 7 studies compared platelet-rich plasma with autologous serum, 5 studies compared platelet-rich plasma with artificial tears, and 5 studies compared autologous serum with artificial tears (Table 1).

### 3.2. Platelet-Rich Plasma Versus Autologous Serum

Table 2 shows the characteristics of the 7 studies discussing PRP and AS.

#### 3.2.1. Schirmer Test Assessment

Across the included studies, six studies evaluated Schirmer test, and their results consistently showed improvement after both AS and PRP treatments, though with varying magnitude and statistical significance. In several studies both AS and PRP led to statistically significant increases in Schirmer scores from baseline, with the PRP groups often showing slightly greater numerical improvement. Özluk et al. reported an increase from 10.9 mm to 13.3 mm in the PRP group and from 7.9 mm to 10.6 mm in the AS group, both highly significant (*p* < 0.001) [31]. Similarly, Wrobel et al. found an increase from 2.95 mm to 5 mm with PRP and from 2.91 mm to 4 mm with AS, again both significant, yet without a statistically significant difference between groups [27].

Jongkhajornpong et al. [30] and Metheetrairut [2] et al. also reported higher mean Schirmer scores in the PRP group compared to the AS group, but without statistical significance between groups. Jongkhajornpong et al. observed post-treatment values of 8.06 mm vs. 6.83 mm (PRP vs. AS) after 4 weeks, while Metheetrairut et al. found a significant between-group improvement, with AS showing a non-significant change.

In contrast, Kang et al. found no significant change in Schirmer I values in either group at any follow-up point, indicating no measurable effect of AS or PRP on tear secretion in their population, despite other ocular surface improvements [28]. Allam et al. demonstrated significant improvement within both groups over the follow-up period (*p* < 0.05), but no statistically significant difference was observed between the groups at the 3-month endpoint [25].

**Figure 2:**
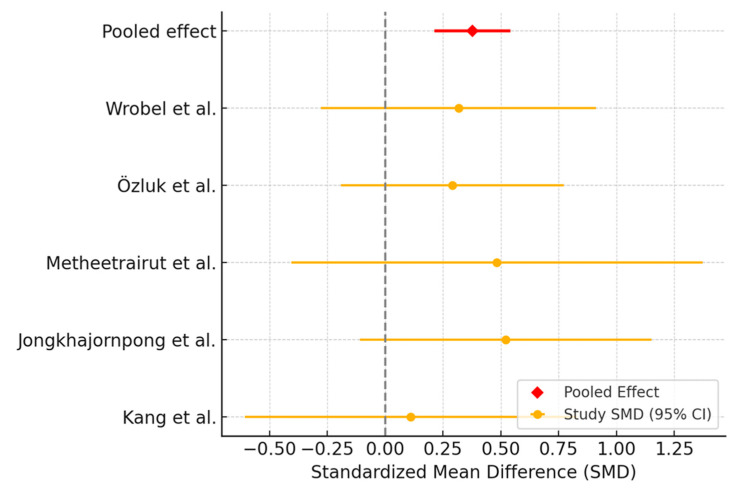
Forest plot showing the effect of interventions (PRP versus AS) on Schirmer’s test.

Five trials were included in the meta-analysis assessing Schirmer test outcomes. The meta-analysis showed that AS was associated with a modest improvement in Schirmer scores compared to PRP. The pooled effect estimate suggested a small but consistent benefit favoring PRP (Figure 2). Heterogeneity analysis indicated moderate variability among the included studies. The Q-statistic was 11.30 (df = 5, *p* = 0.046), and the I^2^ value was 55.8%, suggesting a moderate level of heterogeneity.

#### 3.2.2. Tear Break-Up Time Assessment

Several studies reported improvements in TBUT within both the AS and PRP groups after treatment, without statistically significant differences between the two arms. Kang et al. observed strong intra-group improvements at both 4 and 12 weeks, with PRP and AS showing similar efficacy (no difference at either time point) [28]. Jongkhajornpong et al. reported an increase in TBUT to 4.04 s in the PRP group and 3.90 s in the AS group after 4 weeks, with a mean difference of only 0.14 s (*p* = 0.498) [3]. Likewise, Özluk et al. noted TBUT improvements from 4.3 to 6.7 s in the AS group and from 4.5 to 6.0 s in the PRP group after one month (*p* < 0.001 for both) [31]. Wrobel et al. found increases from 4.72 to 5.0 s in the AS group and from 5.36 to 5.95 s in the PRP group at 3 months, with both intra-group changes being significant but no significant difference between groups (*p* = 0.17) [2].

In another study TBUT also improved modestly after both AS and PRP, but neither change reached statistical significance, likely due to the small sample size. This study contrasts with the above group by showing only a numerical trend rather than clear within-group efficacy [36].

Two studies, Allam et al. and Habib et al., used non-invasive TBUT (NIBUT). Both reported within-group improvements in NIBUT after AS and PRP treatment over three months, with slightly higher mean values at final follow-up in the PRP group in both studies [25,29]. However, neither study found a statistically significant difference between groups at the end of the study period (*p* = 0.167 and *p* = 0.072, respectively).

**Figure 3:**
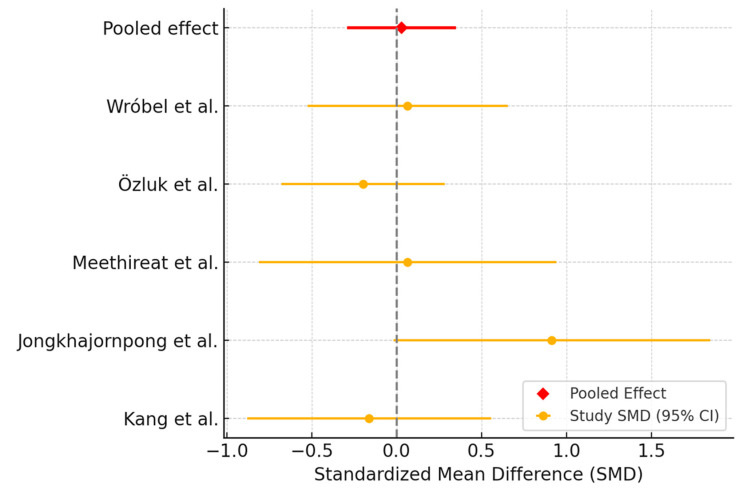
Forest plot showing the effect of interventions (PRP versus AT) on TBUT.

Five trials reported data on TBUT at 1-month follow-up, allowing for meta-analysis. The pooled SMD was 0.03 (95% CI: −0.29 to 0.35), indicating no significant difference (Figure 3). Heterogeneity among studies was low (I^2^ = 13.1%), suggesting consistent effects across trials.

#### 3.2.3. Ocular Surface Disease Index Assessment

Most studies reported improvements in OSDI scores after treatment with both AS and PRP. In nearly all cases, both groups improved significantly from baseline, but between-group differences were either small or statistically non-significant.

A number of studies demonstrated comparable improvement in OSDI scores between PRP and AS groups. Jongkhajornpong et al. reported mean OSDI reductions of −22.22 in the PRP group and −22.47 in the AS group at 4 weeks, meeting their predefined non-inferiority margin (mean difference: 0.24; *p* = 0.9) [30]. Similarly, Kang et al. observed numerical improvements at 12 weeks in both groups, although no statistically significant difference was found at any time point [28]. Metheetrairut et al. showed that both treatments improved symptoms; however, the difference between the two eyes (PRP vs. AS) in the same patient was not statistically significant [26].

A few studies showed substantial symptom relief in both groups, with a slightly greater magnitude of improvement in the PRP arms. Özluk et al. found OSDI scores decreased from 54.1 to 26.8 in the PRP group and from 47.7 to 25.7 in the AS group (*p* < 0.001 for both) [31]. Wrobel et al. observed a significantly greater proportion of patients in the PRP group experiencing OSDI reduction compared to AS (100% vs. 77.3%; *p* = 0.003), and a larger absolute decrease (−19.2 vs. −9.2 points) [24]. Allam et al. also documented statistically significant improvements in both groups over time, but AS-treated patients experienced significantly greater symptom relief at 3 months (*p* < 0.05) [25].

Habib et al. likewise reported symptom improvement in both groups from baseline to 3 months, with numerical advantages for the PRP group at final follow-up (mean OSDI: 22.30 for PRP vs. 25.45 for AS), but these differences were not statistically significant (*p* = 0.183) [29].

**Figure 4:**
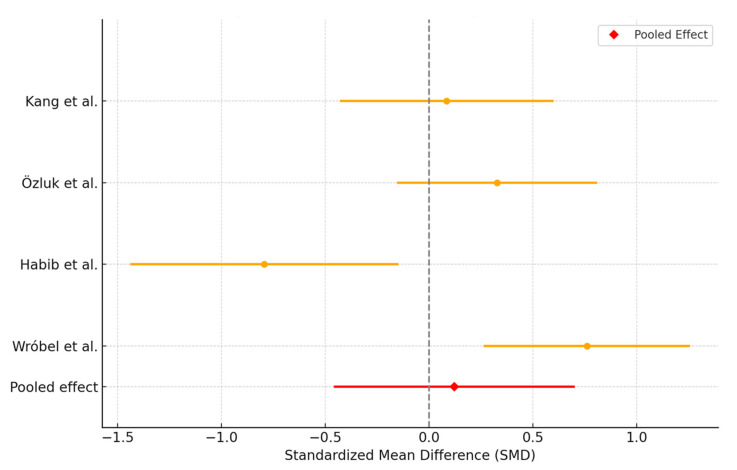
Forest plot showing the effect of interventions (PRP versus AS) on OSDI score.

The meta-analysis included data from four clinical studies. All studies assessed changes in OSDI at one month after treatment initiation. The overall pooled effect size was −0.12 with a 95% CI of −0.46 to 0.70, indicating a non-significant trend toward improvement in OSDI with PRP compared to AS (Figure 4). Heterogeneity analysis revealed a Cochran’s Q of 15.24 (df = 3), corresponding to a highly significant *p*-value and an I^2^ of 80.3%, suggesting substantial variability across studies.

### 3.3. Platelet-Rich Plasma Versus Artificial Tears

Table 3 shows the characteristics of the 5 studies discussing PRP and AT.

#### 3.3.1. Ocular Surface Disease Index Assessment

Four studies evaluated the effect of PRP compared to AT on dry eye symptoms using OSDI. Across all studies, PRP led to greater improvements in OSDI scores than AT, with statistically significant differences consistently reported in favor of PRP.

In the study of Rawat et al., both groups experienced significant OSDI reduction over 3 months, but the mean improvement was substantially greater in the PRP group (−33.4 vs. −8.1 points). The between-group difference at follow-up was −22.7 points in favor of PRP (*p* < 0.0001) [34].

Sachan et al. also demonstrated a progressive and significantly greater reduction in OSDI scores in the PRP group at all time points (1 week, 1 month, and 3 months). At 3 months, the PRP group had a mean OSDI of 38.90 compared to 61.55 in the AT group (*p* = 0.0005), again indicating a clinically meaningful benefit of PRP [36].

García-Conca et al. reported significant reductions in OSDI at both 15 and 30 days after initiating treatment. The PRP group had a mean OSDI change of −24.86 at day 30 versus −5.56 in the AT group (*p* = 0.001) [32]. In the intervention group of another study, OSDI scores significantly improved from 49.38% to 23.35% by day 90 (*p* < 0.001), while the control group showed no meaningful change (42.95% to 44.60%, *p* = 0.083). The between-group difference was also highly significant (*p* < 0.001) [33].

In the comparison between PRP and AT for dry eye disease, three trials were included in the meta-analysis evaluating OSDI score as an outcome. All studies reported greater improvement in OSDI scores in the PRP group compared to AT after treatment. However, the timing of outcome assessment varied slightly, with García-Conca et al. evaluating results at 4 weeks, while the other studies assessed outcomes at 3 months.

**Figure 5:**
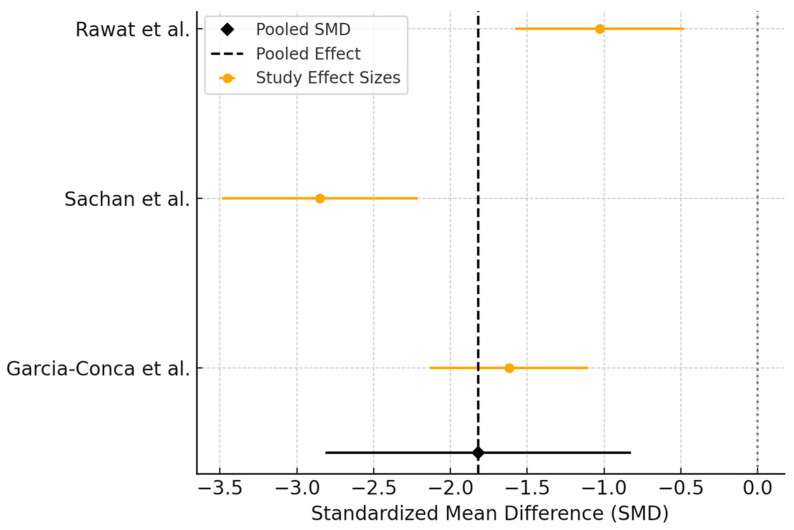
Forest plot showing the effect of interventions (PRP versus AT) on OSDI score.

The pooled SMD was −1.82 [95% CI: −2.86 to −0.77], favoring PRP and indicating a large effect size in reducing OSDI scores compared to AT (Figure 5). This result was statistically significant. However, statistical heterogeneity was substantial across studies (I^2^ = 87%, *p* < 0.001), suggesting considerable variability in treatment effects.

#### 3.3.2. Tear Break-Up Time Assessment

Four studies assessed TBUT, and one study evaluated NIBUT. Across all studies, PRP treatment resulted in significantly greater improvement in tear film stability compared to AT. In the study of Rawat et al., mean TBUT increased significantly in the PRP group from 2.45 ± 2.26 to 4.91 ± 2.46 s (*p* < 0.001), while in the AT group the increase from 2.91 ± 1.48 to 3.03 ± 1.5 s was not statistically significant (*p* = 0.058). The between-group difference at 3 months favored PRP with a mean difference of 1.8 s (*p* < 0.001) [34].

Sachan et al. reported a similar trend: both groups started with comparable TBUT values at baseline (~3.6–3.8 s), but by 3 months, TBUT increased to 7.28 ± 1.66 s in the PRP group versus 5.05 ± 1.22 s in the AT group (*p* = 0.0005). The difference was already evident at 1 month and remained highly significant thereafter [36].

In García-Conca et al., TBUT improved modestly in both groups, but the change in the PRP group was greater. At 30 days, the mean increase in TBUT for the left eye was 0.6 s in the PRP group compared to a decrease of 0.1 s in the AT group (*p* = 0.024). While this absolute difference is small, it was statistically significant [32]. The study of Elessawy et al. [33] showed slight improvement in both groups: from 5.57 ± 3.26 s to 6.35 ± 3.2 s in the intervention group (*p* = 0.09) and from 5.50 ± 3.26 s to 6.35 ± 3.1 s in the control group (*p* = 0.08). The difference between groups was not statistically significant (*p* = 0.2).

Mohammed et al. used NIBUT and reported significantly greater improvement in the PRP-treated eye at 1, 2, and 3 months compared to the control (AT-treated) eye, with *p* < 0.001 at all timepoints [35].

**Figure 6:**
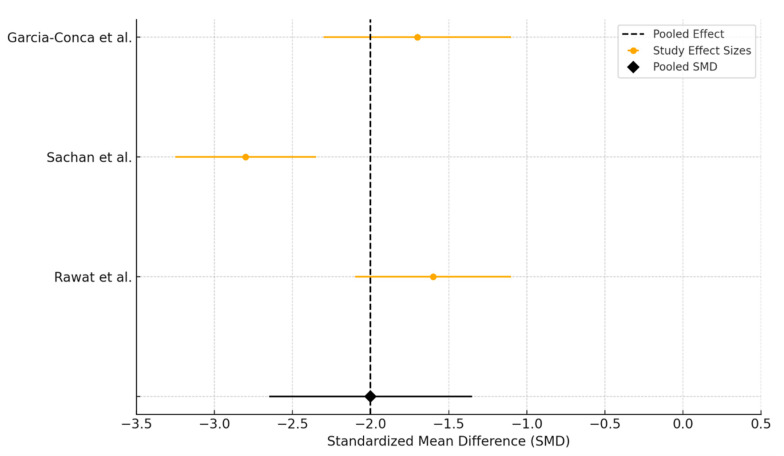
Forest plot showing the effect of interventions (PRP versus AT) on TBUT.

Three trials assessed the impact of PRP versus AT on TBUT at three months, including data from Rawat et al., Sachan et al., and Garcia-Conca et al. All studies reported improvements in TBUT following treatment, with PRP showing a consistently greater increase compared to AT. Standardized mean differences favored PRP across all trials, and the pooled analysis demonstrated a significant benefit of PRP over AT in enhancing tear film stability (Figure 6). However, the results were accompanied by considerable heterogeneity. The I^2^ value was 91.7%, with a Q statistic of 24.08 (df = 2, *p* < 0.0001), indicating substantial between-study variability beyond chance.

#### 3.3.3. Schirmer Test Assessment

Five studies reported Schirmer test outcomes, evaluating the effect of PRP versus artificial tears on aqueous tear production. Among these, four studies showed statistically significant improvements in the PRP groups, while one reported no significant difference.

In the study of Rawat et al., Schirmer scores remained nearly unchanged over the 3-month period in both groups. The PRP group improved marginally from 4.14 mm to 4.19 mm (*p* = 0.083), while the AT group increased from 4.26 mm to 4.30 mm (*p* = 0.159). The between-group difference was also not significant (*p* = 0.44) [34].

Sachan et al. reported highly significant improvements in the PRP group at all time points. At 3 months, Schirmer scores increased from 4.3 mm to 9.7 mm in the PRP group, compared to 3.2 mm to 5.2 mm in the AT group (*p* < 0.0005) [36]. Elessawy et al. a significant improvement in the intervention group, increasing from 4.00 ± 2.13 mm to 5.24 ± 3.60 mm (*p* = 0.011), while the control group showed no meaningful change (3.31 ± 1.85 mm to 3.14 ± 1.90 mm, *p* = 0.714). The between-group difference was statistically significant (*p* = 0.005) [33].

Mohammed et al. found statistically significant improvement in Schirmer scores in the PRP-injected eye compared to the control eye at 1, 2, and 3 months (all *p* < 0.001) [35].

García-Conca et al. also documented statistically significant improvements in Schirmer values at both 15 and 30 days. At 30 days, the PRP group showed a mean increase of 1.9 mm versus only 0.3/0.5 mm in the AT group (*p* = 0.002 for RE; *p* = 0.001 for LE) [32].

**Figure 7:**
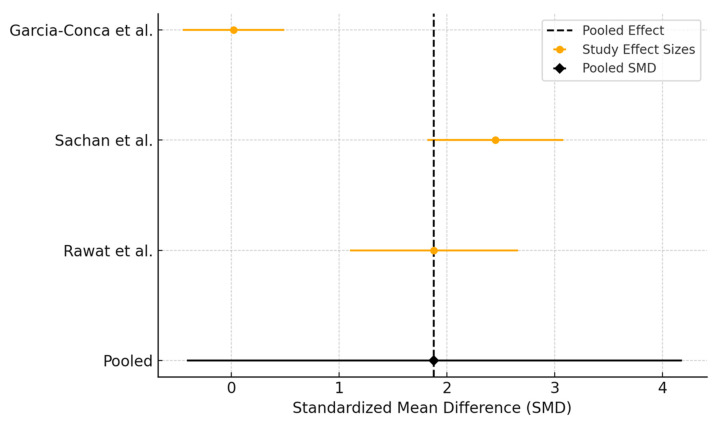
Forest plot showing the effect of interventions (PRP versus AT) on Schirmer’s test.

Three studies assessed the efficacy of PRP compared to AT in improving Schirmer test scores after 3 months of treatment (Figure 7). Sachan et al. demonstrated a large improvement in tear production in favor of PRP, while Garcia-Conca et al. reported a moderate benefit. In contrast, Rawat et al. found minimal difference between the two groups. The pooled SMD was 1.88 (95% CI: −0.41 to 4.18), indicating a favorable trend toward PRP. Very high heterogeneity was detected (Q = 55.19, I^2^ = 96.4%), most probably from differences in baseline tear secretion or patient populations.

### 3.4. Autologous Serum Versus Artificial Tears

Table 4 shows the characteristics of the 5 studies discussing AS and AT.

Five studies comparing AS with AT in DED were included. Three of the studies utilized a crossover design, while the other two were parallel-group randomized trials. The follow-up durations across studies ranged from two to twelve weeks. A meta-analysis was not possible, because of the crossover design with short follow-up durations of several trials, and because standard deviations were not consistently reported, which precluded weighted quantitative synthesis. All studies enrolled patients with moderate to severe DED, with some including those with Sjögren’s syndrome.

In terms of symptom improvement assessed by OSDI, all five studies showed greater benefit with AS compared to AT. Urzúa et al. observed a mean OSDI reduction from 59 ± 10 to 30 ± 8 in the AS group, compared to a reduction from 51 ± 7 to 41 ± 8 with AT, corresponding to a mean difference of approximately −11 points in favor of AS (*p* = 0.002) [38]. Celebi et al. also reported a statistically significant reduction in OSDI of about −18.6 points with AS (*p* < 0.001) [39]. Yilmaz et al. found that while both AS and AT improved OSDI scores over time, the reductions were consistently greater during AS treatment periods [40]. Zheng et al. demonstrated a between-group difference in OSDI of −10.3 points (95% CI: −13.6 to −7.0, *p* < 0.001), with AS treatment showing a mean change of −26.4 ± 9.8 compared to −16.1 ± 8.9 with PFAT at 12 weeks [41]. Kojima et al. did not report OSDI directly but used a visual analog symptom score, with significantly better outcomes observed in the AS group (VAS: 52 ± 24 versus 70 ± 20, *p* < 0.05) [37].

Regarding tear film stability, improvements in TBUT were reported in all studies that measured it. Urzúa et al. noted an increase from 4 to 6 s with AS and from 3 to 4 s with AT, although this difference was not statistically significant [38]. Celebi et al. documented a significant increase in TBUT with AS (*p* < 0.001) [3]. Zheng et al. reported a TBUT improvement of +2.4 ± 1.3 s in the ASED group versus +0.6 ± 0.9 s with AT (*p* < 0.001). Similarly, Kojima et al. reported an increase from 2.3 ± 2.3 to 4.3 ± 2.6 s in the AS group, significantly better than AT (*p* < 0.05) [37]. Other outcomes also favored AS. Zheng et al. reported an increase in Schirmer test values of +4.6 ± 2.4 mm in the AS group compared to +1.5 ± 1.8 mm with AT (*p* < 0.001) [38], while Kojima et al. observed no significant difference [37].

### 3.5. Subgroup Analysis

#### 3.5.1. Outcome Analysis Depending on Route of Administration in Platelet-Rich Plasma Versus Artificial Tears

At three months post-treatment, the greatest improvement in Schirmer test scores was observed in the study by Mohammed et al., where PRP was administered via lacrimal gland injection [35]. Tear production increased significantly from a baseline of 5.14 ± 1.29 mm to 12.64 ± 0.93 mm, representing a mean change of +7.5 mm (*p* < 0.001). Another randomized controlled study involving transcutaneous PRP injection into the lacrimal gland also demonstrated a significant but more modest increase, from 4.00 ± 2.13 mm to 5.24 ± 3.60 mm (*p* = 0.011), representing a +1.24 mm gain [33]. Among the studies using PRP eye drops, Sachan et al. also reported a significant increase, from 4.3 ± 2.1 mm to 9.7 ± 1.1 mm at three months (*p* < 0.0005), resulting in a mean change of +5.4 mm [36]. In contrast, Rawat et al. found no meaningful change in Schirmer scores, with values increasing only marginally from 4.14 ± 2.50 mm to 4.19 ± 2.48 mm (*p* = 0.083) [34].

#### 3.5.2. Outcome Analysis Depending on Route of Administration in Platelet-Rich Plasma Versus Autologous Serum

When comparing PRP delivered by injection to PRP applied as eye drops, the injection method demonstrated a more pronounced and consistent improvement in both subjective symptoms and tear production at 3 months. In the study of Allam et al., PRP injection reduced OSDI by 13.25 points and improved Schirmer I scores by 6.20 mm, both statistically significant [25]. In contrast, Kang et al. reported minimal changes in either outcome with eye drops, and the results were not significant [28]. The studies of Wróbel-Dudzińska et al. [27] and Habib et al. [29] showed greater improvements with eye drops, including an OSDI reduction of 19.19 and 13.85 points, respectively, and a +2.05 mm increase in Schirmer, though not matching the increase seen with injection in Allam’s study.

### 3.6. Risk of Bias

The risk of bias assessment using the ROBINS-I tool included five non-randomized studies. Three studies were judged to have an overall moderate risk of bias, while two had a low-to-moderate risk. The most frequent concerns were related to confounding and classification of interventions (Table 5).

For randomized controlled trials assessed with the RoB 2 tool (n = 10), seven studies were rated as having low risk of bias, while three studies were judged to have some concerns, primarily due to deviations from intended interventions and selective reporting (Table 6). For the three studies with a crossover design, we used the modified Cochrane RoB 2 tool for crossover trials, which includes additional domains to assess carryover effects and adequacy of washout periods.

## 4. Discussion

Tear secretion, evaluated by Schirmer Test, and tear break-up time (TBUT) are fundamental parameters in the evaluation of DED, providing information into both the quantity and functionality of the tear film. These measures are not only standard diagnostic criteria but also serve as primary outcome indicators in clinical trials assessing the efficacy of various treatments [42,43]. Reduced tear production reflects compromised lacrimal gland function and is commonly associated with aqueous-deficient dry eye. TBUT, on the other hand, evaluates the time it takes for the tear film to break up after a blink and serves as a surrogate for tear film stability [44,45,46]. In the assessment of these changes, non-invasive tear break-up time (NITBUT) is generally considered more physiological and reproducible, as fluorescein itself can destabilize the tear film, leading to shorter values and higher variability between examiners. However, TBUT remains the more widely available and commonly used method in clinical trials. In the present review, most studies relied on TBUT [42,43]. The Schirmer test remains one of the most widely used diagnostic tools in DED, as it provides a direct measure of basal and reflex tear secretion. Despite variability and some limitations, it continues to serve as a key outcome measure in clinical trials and is endorsed in international guidelines such as the DEWS II report. Its significance lies not only in diagnosing lacrimal gland dysfunction but also in monitoring treatment efficacy, making it a fundamental parameter in evaluating therapies [47,48].

Importantly, tear film stability is influenced by more than just tear volume [49]. It is highly dependent on the quality of the tear film, which comprises three distinct layers: a superficial lipid layer that prevents evaporation, a middle aqueous layer that provides hydration and nutrients, and a mucin layer that ensures even distribution of tears across the ocular surface [49,50]. Alterations in any of these layers, due to inflammation, glandular dysfunction, or surface damage, can lead to premature tear film breakup, ocular surface irritation, and the hallmark symptoms of DED such as dryness, burning, and visual disturbance [50,51]. Therefore, an ideal therapeutic approach should aim not only to increase tear volume but also to improve tear film composition and restore homeostasis to the ocular surface. Blood-derived therapies such as PRP and AS have shown promise in this regard by delivering growth factors, anti-inflammatory mediators, and other bioactive components that may enhance both tear secretion and tear film integrity [52,53,54].

OSDI is one of the most widely used patient-reported outcome measures in DED research and clinical practice. It is a validated, standardized questionnaire that quantifies the severity of DED symptoms as well as their impact on vision-related quality of life [55,56]. The OSDI complements objective measures like Schirmer test and TBUT by capturing the subjective burden of disease from the patient’s perspective [57,58]. In clinical trials, a reduction in OSDI score is a primary endpoint for determining treatment efficacy, particularly in therapies aimed at improving patient comfort and functional quality of life [19,59].

In addition to the clinical outcomes analyzed, understanding the mechanisms of action of PRP, AS, and AT provides important context for their therapeutic roles in DED. PRP contains a high concentration of platelets and associated bioactive molecules, such as growth factors and anti-inflammatory mediators. These factors promote epithelial regeneration, angiogenesis modulation, and neuroprotection, while simultaneously alleviating oxidative stress on the ocular surface [60,61]. Once activated, PRP provides a sustained release of growth factors that contribute to accelerated wound healing and restoration of tissue homeostasis [62]. AS eye drops mimic the composition of natural tears, providing essential tear components such as vitamins, fibronectin, and growth factors. They are thought to alleviate dry eye primarily through supplying trophic and anti-oxidative elements to the ocular surface [63]. AS eye drops may also contain pro-inflammatory cytokines derived from leukocytes and monocytes. In addition, the uncontrolled presence of immunoglobulins and complement factors could be harmful for certain patients, particularly those with underlying immunological disorders [13]. In addition, technologies related to plasma rich in growth factors demonstrate that activation of platelets within AS preparations can create a biocompatible fibrin scaffold, ensuring a progressive release of growth factors that support wound healing and tissue repair [13]. Artificial tears primarily act by supplementing tear volume and improving tear film stability [64]. Their formulations often include viscosity-enhancing agents, electrolytes, osmoprotectants, and antioxidants. Artificial tears also differ widely in their osmolarity, viscosity, and electrolyte composition [13]. Recent evidence suggests certain ingredients may also play roles in modulating wound healing and inflammation [64,65]. Furthermore, beyond their role in dry eye disease, artificial tears have a broad therapeutic spectrum, including management of anterior eye trauma, infection, inflammation, and contact lens-related complications [5]. This mechanism might give an explanation in why PRP and AS offer advantages over conventional AT by delivering not only lubrication but also active biological factors that support regeneration and repair of the ocular surface.

This systematic review synthesizes evidence from trials evaluating autologous blood-derived therapies for dry eye disease, with a focus on PRP, AS, and their comparison with AT. The findings confirm that both PRP and AS are generally more effective than AT in improving clinical symptoms and ocular surface parameters. Additionally, this review highlights a potentially important clinical consideration: the route of administration may influence therapeutic outcomes. Although most studies reported that blood-derived eye drops were well tolerated, adverse events were only minimally described. Beyond clinical efficacy, additional factors deserve consideration when evaluating the role of blood-derived therapies for DED. The preparation of autologous serum and PRP requires specialized laboratory facilities, trained personnel, and repeated venipuncture, all of which may influence feasibility in daily practice and place demands on healthcare resources. Economic analyses would therefore be essential to determine whether the improvements observed in tear film parameters and symptoms translate into cost-effective value for patients and health systems. Equally important is the patient perspective. Most available studies relied exclusively on symptom scales such as the OSDI or visual analog scores, which, while valuable, provide only a partial view of the treatment impact. Broader patient-reported outcomes, such as satisfaction with therapy, adherence, functional vision, and overall quality of life, remain largely unexplored.

While most included studies demonstrated statistically significant improvements in parameters, the clinical meaningfulness of these changes is more nuanced. Elessawy et al. reported a statistically significant increase in Schirmer values with PRP injection; however, this represents a mean gain of only ~1 mm which is well below the generally accepted threshold of ≥5 mm considered clinically meaningful in DED [47,48]. Similarly, García-Conca et al. found a statistically significant improvement of 0.6 s in TBUT after PRP, but this is far less than the 2 s increase typically regarded as clinically relevant [66]. OSDI improvements exceeding 20 points as seen in a few of the trials surpass the minimal clinically important difference (MCID) of approximately 10 points and are therefore likely to reflect meaningful symptom relief and better daily functioning [67]. This discrepancy between statistical and clinical significance probably reflects the heterogeneity of patient populations, high variability in tests such as Schirmer and TBUT, and differences in follow-up duration and sample size across studies. In some cases, large standard deviations mean that small numerical changes achieve statistical significance but remain clinically trivial, while in others, improvements clearly surpass accepted MCIDs and can be considered relevant for patient-centered care.

Several traditional meta-analyses have examined the efficacy of autologous blood-derived therapies in DED, yet these have often been limited in scope, focusing on either AS or PRP alone. For example, Wang et al. [68] and Quan et al. [69] conducted pairwise meta-analyses comparing AS to artificial tears, consistently demonstrating the superior efficacy of AS in improving clinical parameters such as OSDI, TBUT, and corneal staining. Similarly, Akowuah et al. [61] reported symptomatic and functional improvement with PRP, although their analysis was primarily based on observational and self-controlled studies. In contrast, our systematic review consolidates and compares evidence across a wider spectrum of blood-derived interventions, including AS, PRP (both topical and injected), and their comparative efficacy against artificial tears. Zhang et al. conducted a network meta-analysis of 16 randomized controlled trials comparing six different blood-derived treatments for dry eye disease, evaluating their relative efficacy in improving clinical outcomes such as Schirmer’s I, TBUT, CFSS, and OSDI [70]. Furthermore, our subgroup synthesis highlights clinically relevant information: the route of administration may influence the therapeutic impact of PRP, with lacrimal gland injection showing superior improvements in tear secretion (Schirmer test) compared to PRP eye drops.

### Advantages and Limitations

This systematic review offers several important strengths. First, it provides a comprehensive comparison of blood-derived therapies across a variety of administration routes and formulations. It specifically explores the impact of PRP delivery method, injection versus eye drops, on treatment outcomes, offering new clinical insight that may guide future therapeutic strategies. Finally, the inclusion of multiple outcome measures (subjective symptoms and objective tear function) allows for a multidimensional assessment of efficacy.

Nonetheless, several limitations must be acknowledged. An important issue identified in this review is the substantial heterogeneity across the included studies. Patient populations varied widely, and disease severity was also inconsistent, ranging from moderate to very severe forms, which likely influenced baseline tear parameters and responsiveness to treatment.

Equally important, the preparation protocols differed considerably. Autologous serum was prepared at concentrations ranging from 20% to 100%, with variable dilution solutions and storage conditions, while PRP protocols showed marked variation in centrifugation steps, platelet enrichment levels, and whether activation was performed prior to administration. Routes of administration were also diverse, from topical instillation to direct lacrimal gland injection, with corresponding differences in dosing frequency and treatment duration. This degree of heterogeneity complicates direct comparability of results and reduces the precision of pooled estimates in this systematic review. It highlights the urgent need for standardized protocols in trial design, preparation of blood-derived products, and outcome reporting. Harmonization in these areas would enhance reproducibility, enable higher-quality meta-analyses, and ultimately provide clearer guidance for clinical practice.

Second, variability in blinding, masking, and reporting standards introduces potential risk of bias. Most trials had small sample sizes and short follow-up durations, limiting the ability to assess long-term safety and efficacy. A key limitation of this review is that a meta-analysis for the AS versus AT comparison could not be performed due to the crossover design and incomplete data reporting in several trials. As a result, conclusions about the superiority of AS over AT rely mainly on descriptive synthesis rather than pooled estimates, which reduces the strength of the evidence.

Another important consideration is the current lack of international standards for the preparation of PRP and AS in the context of DED. Significant heterogeneity exists among studies in terms of centrifugation protocols, dilutions, and routes of administration. This variability not only limits comparability but also makes it difficult to draw firm conclusions about the superiority of one formulation or route over another. Moreover, while most short-term trials report favorable outcomes and good tolerability, the safety of repeated or injectable PRP requires further investigation in larger studies with longer follow-up. The durability of therapeutic effects beyond 3–6 months is rarely reported, and the potential risks remain poorly characterized.

Future research should prioritize the development and adoption of standardized preparation protocols and rigorously assess both efficacy and safety over extended periods (≥6–12 months).

## 5. Conclusions

This systematic review suggests that both PRP and AS may provide benefits over AT in improving symptoms and tear film parameters in DED. PRP could offer certain advantages over other treatments, particularly when administered through injections, but findings remain inconsistent across studies. Considerable variability in preparation methods and study designs highlights the importance of standardization. Overall, blood-derived treatments represent promising therapeutic approaches for refractory DED, yet further well-designed trials are required to clarify their relative efficacy, ensure long-term safety, and establish international standards for preparation and administration.

## Figures and Tables

**Table 1:** Characteristics of the included studies.

Study	Year	Country	Comparison	Sample Size	Age	Outcome	Included in Meta-Analysis
Allam et al. [25]	2021	Egypt	PRP vs. AS	20/20	56.0 ± 8.2	OSDI score, Schirmer test, CFS, TMH, NIBUT, LLT	No
Metheetrairut et al. [26]	2022	Thailand	PRP vs. AS	10/10	65.6 ± 14.35	OSDI score, CFS, TBUT, Schirmer test, BCVA, IOP	Yes
Wróbel-Dudzińska et al. [27]	2023	Poland	PRP vs. AS	22/22	62.2 ± 8.5/62.9 ± 10.9	OSDI score, BCVA, Schirmer test, TBUT, CFS, meibomian gland parameters	Yes
Kang et al. [28]	2023	South Korea	PRP vs. AS	14/16	54.07 ± 11.28/54.56 ± 11.93	OSDI score, CFS, CDVA, Schirmer test, TBUT	Yes
Habib et al. [29]	2023	Egypt	PRP vs. AS	20/20	N/A	OSDI score, CFS, CDVA, Schirmer test, NIBUT	Yes
Jongkhajornpong et al. [30]	2024	Thailand	PRP vs. AS	48/48	N/A	OSDI score, Oxford scale, TBUT Schirmer test, meibum quality, adverse effects	Yes
Ozluk et al. [31]	2024	Turkey	PRP vs. AS	34/33	55.1 ± 19.7/65.7 ± 12.3	BCVA, Schirmer test, OSDI score, TBUT, Oxford scale	Yes
Garcia Conca et al. [32]	2018	Spain	PRP vs. AT	44/39	64 ± 11.2	OSDI score, osmolarity, Schirmer test, TBUT, CFS	Yes
Elessawy et al. [33]	2021	Egypt	PRP vs. AT	20/20	54 ± 10/51 ± 6	Schirmer test, TBUT, OSDI score, CFS, CIC	No
Rawat et al. [34]	2022	India	PRP vs. AT	31/30	52.8 ± 12.8/55.5 ± 13.4	Schirmer test, TBUT, OSDI score, CFS	Yes
Mohammed et al. [35]	2022	Egypt	PRP vs. AT	14/14	43.4 ± 7.85	TMH, NITBUT, LLT, Schirmer test, CFS, meiboscore	No
Sachan et al. [36]	2025	India	PRP vs. AT	20/20	51 ± 14/50 ± 17	OSDI score, BCVA, TMH, TBUT, Schirmer test, CFS, CIC	Yes
Kojima et al. [37]	2005	Japan	AS vs. AT	10/10	62.3 ± 12.5/65.4 ± 9.7	Schirmer test, TBUT, CFS	No
Urzua et al. [38]	2012	Chile	AS vs. AT	12/12	52 ± 6.3	OSDI score, CFS, TBUT	No
Celebi et al. [39]	2014	Turkey	AS vs. AT	20/20	56.05 ± 8.07	OSDI score, TBUT, Schirmer test, Oxford scale	Yes
Yilmaz et al. [40]	2017	Turkey	AS vs. AT	24/24	25 ± 4.02	BCVA, IOP, TBUT, Schirmer test, OSDI score	No
Zheng et al. [41]	2023	China	AS vs. AT	116/116	54.6 ± 12.4/55.2 ± 12.4	OSDI score, TBUT, Schirmer test, CFS, CIC	Yes

Abbreviations: AS—autologous serum; AT—artificial tears; BCVA—best corrected visual acuity; CFS—corneal fluorescein staining; CIC—conjunctival impression cytology; IOP—intraocular pressure; LLT—lipid layer thickness; N/A—not available; NIBUT—non-invasive break-up time; OSDI—ocular surface disease index; PRP—platelet rich plasma; TBUT—tear break-up time; TMH—tear meniscus height.

**Table 2:** Characteristics of the studies comparing PRP and AS in patients with dry eye disease.

Study	Sample Size	Treatment Method	Preparation/Administration	Therapeutic Dose	Follow-Up	Side Effects
Allam et al. [25]	20/20	AS: eye drops PRP: injection into lacrimal gland	AS: 20 mL whole blood → clot → centrifugation → diluted 50% with 0.9% saline PRP: 10 mL blood with sodium citrate → centrifugation → activated with CaCl, injected transcutaneously into lacrimal gland area	AS: 5×/day for 12 weeks PRP: 1 mL (days 0, 30, 60, 90)	1 month/2 months/3 months	mild ocular irritation in 4 eyes (AS group)
Metheetrairut et al. [26]	10/10	AS/PRP: eye drops	AS: 35 mL blood incubated 2 h at 31 °C; centrifuged at 3000× *g* for 30 min; diluted to 20% with BSS PRP: 35 mL blood with sodium citrate; centrifuged at 2200× *g* for 10 min; diluted to 20% with balanced salt solution	AS/PRP: instilled hourly for 1 month	1 month	None reported
Wróbel-Dudzińska et al. [27]	22/22	AS/PRP: eye drops	AS: 5 mL blood without anticoagulant; clotting 1 h; centrifuged twice at 3000 rpm ×10 min; filtered; dispensed undiluted PRP: 9 mL blood with sodium citrate; centrifuged 1400 rpm ×10 min (no platelet activation); platelet-rich top layer collected (avoiding buffy coat); dispensed undiluted	AS/PRP: instilled 1 drop ×5/day for 3 months	1 month/3 months	None reported
Kang et al. [28]	14/16	AS/PRP: eye drops	AS: 24 mL blood → clot 2 h → centrifuged 3500× *g* for 15 min → diluted to 20% with sodium hyaluronate PRP: 22 mL blood + sodium citrate → processed with PRS Bio Kit; centrifuged 3000× *g* for 3 min → buffy coat + plasma mixed → diluted to 20% with sodium hyaluronate	AS/PRP: instilled 1 drop, 6× daily for 12 weeks	1 month/3 months	None reported
Habib et al. [29]	20/20	AS/PRP: eye drops	Not reported	AS/PRP: instilled regularly over 3 months	1 month/3 months	None reported
Jongkhajornpong et al. [30]	48/48	AS/PRP: eye drops	AS: blood allowed to clot 1–2 h → centrifuged at 3000× *g* for 15 min → serum filtered → dispensed undiluted (100%) PRP:Blood anticoagulated with sodium citrate (9:1 ratio), centrifuged at 350× *g* for 10 min → plasma fraction collected as APRP → dispensed undiluted (100%)	AS: 1 drop/eye every 2 h between 08:00–22:00 (≈8×/day) for 4 weeks.	2 weeks/4 weeks	None reported
Ozluk et al. [31]	34/33	AS/PRP: eye drops	Not reported	AS/PRP: instilled regularly over one month	1 month	None reported

**Table 3:** Characteristics of the studies comparing PRP and AT in patients with dry eye disease.

Study	Sample Size	Treatment Method	Components/Composition	Preparation/Administration	Therapeutic Dose	Follow-Up	Side Effects
Garcia Conca et al. [32]	44/39	PRP/AT: eye drops	AT: Hypotonic aqueous solution of SH 0.18% + ions (Vismed^®^, TRB Chemedica, Newcastle-under-Lyme, UK)	PRP: Blood (40 mL) collected → centrifuged; pellet resuspended → ~4.5 mL PRP/tube.	PRP: 1 drop every 3 h, 6×/day, for 30 days. AT: instilled regularly	2 weeks/4 weeks	None reported
Elessawy et al. [33]	20/20	AT: eye drops PRP: injection into lacrimal gland	AT: Preservative-free (Refresh Plus^®^, Allergan, Dublin, Ireland; carboxymethylcellulose-based)	PRP: 10 mL whole blood → 1st spin 160 g ×10 min → plasma + buffy coat transferred → 2nd spin 160 g ×15 min → PRP layer collected; 0.1 mL CaCl_2_ added	AT: instillation 3× daily for 3 months PRP: injection repeated at day 0, 30, 60	3 months	PRP: 1 ocular infection, 2 vasovagal episodes, 2 ecchymoses, 5 transient periorbital edemas
Rawat et al. [34]	31/30	PRP/AT: eye drops	AT:0.5% Carboxymethylcellulose (CMC) solution	PRP: Blood collected in citrated tubes → centrifuged per Alió 2012 protocol → platelet-rich plasma fraction aspirated → diluted 1:4 with BSS	AT/PRP: 4–6×/day, for 3 months	1 week/2 weeks/1 month/3 months	None reported
Mohammed et al. [35]	14/14	AT: eye drops PRP: injection into lacrimal gland	AT: preservative-free lubricants	PRP: 10 mL blood in sodium citrate → centrifuged → platelet-rich fraction collected → activated with 10% CaCl_2_.	AT: instilled regularly PRP: 1 mL PRP injected transcutaneously into lacrimal gland	1 month/2 months/3 months	None reported
Sachan et al. [36]	20/20	PRP/AT: eye drops	AT: Systane Complete (propylene glycol 0.6%, Alcon, Geneva, Switzerland)	PRP: 10 mL blood in sodium citrate → soft spin → supernatant spun→ 3–5 mL PRP extracted → diluted to 20% with BSS.	AT/PRP: 1 drop, 4–6×/day, for 3 months	1 week/1 month/3 months	None reported

**Table 4:** Characteristics of the studies comparing AS and AT in patients with dry eye disease.

Study	Sample Size	Treatment Method	Components/Composition	Preparation/Administration	Therapeutic Dose	Follow-Up	Side Effects
Kojima et al. [37]	10/10	AS/AT: eye drops	AT: Preservative-free lubricant	AS: 40 mL venous blood → centrifuged 1500 rpm, 5 min → serum separated → diluted to 20% saline.	AS/AT: 1 drop, 6×/day, both eyes, for 2 weeks	2 weeks	None reported
Urzua et al. [38]	12/12	AS/AT: eye drops	AT: Systane (propylene glycol 0.6%, Alcon)	AS: 20 mL blood → clotting 2 h → centrifugation → supernatant serum collected → diluted 20%	AS/AT: 1 drop 4×/day for 14 days.	1 month/2 months	None reported
Celebi et al. [39]	20/20	AS/AT: eye drops	AT: Refresh^®^ Preservative-Free AT (Allergan, carboxymethylcellulose and lubricants)	AS: 20 mL venous blood → clot 2 h RT → centrifugation → ~5 mL serum collected → diluted 20%	AS/AT: 1 drop, 4×/day, for 1 month	1 month/2 months	None reported
Yilmaz et al. [40]	24/24	AS/AT: eye drops	AT: Preservative-free lubricant	AS: 20 mL venous blood → clot 2 h → centrifuge → supernatant serum collected → diluted 40% saline	AS/AT: 1 drop, 4×/day, for 1 month.	1 month/2 months	None reported
Zheng et al. [41]	116/116	AS/AT: eye drops	AT: Preservative-free 0.1% sodium hyaluronate (Hylo-Comod^®^, Saarbrücken, Germany).	AS: 40 mL venous blood → centrifugation → serum separated, diluted to 20% with saline	AS/AT: 1 drop/eye, 4× daily, for 12 weeks.	3 months	AS/AT: Mild, transient ocular irritation/redness/itching/discharge

**Table 5:** Risk of bias assessment using ROBINS I tool.

Study	D1	D2	D3	D4	D5	D6	D7	Overall
Rawat et al. [34]								Moderate
Sachan et al. [36]								Moderate
Mohammed et al. [35]								Low/Moderate
Ozluk et al. [31]								Moderate
Wróbel-Dudzińska et al. [27]								Low/Moderate

Legend: Green = Low risk of bias; Yellow = Moderate risk of bias/some concerns. Domains: D1—Bias due to confounding; D2—Bias in selection of participants into the study; D3—Bias in classification of interventions; D4—Bias due to deviations from intended interventions; D5—Bias due to missing data; D6—Bias in measurement of outcomes; D7—Bias in selection of the reported result.

**Table 6:** Risk of bias assessment using Rob2 tool.

Study	D1	D2	D3	D4	D5	D6	Overall
Garcia-Conca et al. [32]							Low/Moderate
Allam et al. [25]							Low/Moderate
Metheetrairut et al. [26]							Low
Yilmaz et al. [40]							Low
Celebi et al. [39]							Low
Urzua et al. [38]							Low
Kojima et al. [37]							Moderate
Zheng et al. [41]							Low
Kang et al. [28]							Low
Jongkhajornpong et al. [30]							Low

Legend: Green = Low risk of bias; Yellow = Moderate risk of bias/some concerns; Grey = not applicable. Domains: D1—Bias arising from the randomization process; D2—Bias due to deviations from intended interventions; D3—Bias due to missing outcome data; D4—Bias in measurement of the outcome; D5—Bias in selection of the reported result; D6—Bias arising from period and carryover effects.

## Data Availability

No new data created.
